# Co-localization and functional cross-talk between A_1_ and P2Y_1_ purine receptors in rat hippocampus

**DOI:** 10.1111/j.1460-9568.2007.05697.x

**Published:** 2007-08

**Authors:** I. Tonazzini, M. L. Trincavelli, J. Storm-Mathisen, C. Martini, L. H. Bergersen

**Affiliations:** 1 Department of Psychiatry Neurobiology Pharmacology and Biotechnology, University of Pisa via Bonanno 6, 56126, Pisa, Italy; 2 Department of Anatomy, IMB, and Centre for Molecular Biology and Neuroscience (CMBN), University of Oslo PO Box 1105, Blindern, NO-0317, Oslo, Norway

**Keywords:** adenosine, ATP, brain damage, electron microscopy, G protein coupled receptors, immunocytochemistry

## Abstract

Adenosine and ATP, via their specific P1 and P2 receptors, modulate a wide variety of cellular and tissue functions, playing a neuroprotective or neurodegenerative role in brain damage conditions. Although, in general, adenosine inhibits excitability and ATP functions as an excitatory transmitter in the central nervous system, recent data suggest the existence of a heterodimerization and a functional interaction between P1 and P2 receptors in the brain. In particular, interactions of adenosine A_1_ and P2Y_1_ receptors may play important roles in the purinergic signalling cascade. In the present work, we investigated the subcellular localization/co-localization of the receptors and their functional cross-talk at the membrane level in Wistar rat hippocampus. This is a particularly vulnerable brain area, which is sensitive to adenosine- and ATP-mediated control of glutamatergic transmission. The postembedding immunogold electron microscopy technique showed that the two receptors are co-localized at the synaptic membranes and surrounding astroglial membranes of glutamatergic synapses. To investigate the functional cross-talk between the two types of purinergic receptors, we evaluated the reciprocal effects of their activation on their G protein coupling. P2Y_1_ receptor stimulation impaired the potency of A_1_ receptor coupling to G protein, whereas the stimulation of A_1_ receptors increased the functional responsiveness of P2Y_1_ receptors. The results demonstrated an A_1_–P2Y_1_ receptor co-localization at glutamatergic synapses and surrounding astrocytes and a functional interaction between these receptors in hippocampus, suggesting ATP and adenosine can interact in purine-mediated signalling. This may be particularly important during pathological conditions, when large amounts of these mediators are released.

## Introduction

ATP and adenosine, via their specific P2 and P1 purinergic receptors (Fredholm *et al*., 1994), mediate a wide variety of physiological processes including neuromodulation and neurotransmission. Moreover, the purinergic system has been implicated in many pathological and neurodegenerative conditions, in which massive release of ATP and, in turn, ADP and adenosine production, occur from damaged or dying cells, i.e. following ischemia, necrosis or trauma (Rathbone *et al*., 1999; Burnstock, 2004). Several reports have described a dualistic neuroprotective-neuromodulatory role of ATP interacting with the specific ionotropic receptors (P2XR) and G protein coupled receptors (GPCRs; P2YR) (Fredholm *et al*., 1994). Among P2YR, P2Y_1_ receptors (P2Y_1_R) appear to be of particular interest in patho-physiological mechanisms with both detrimental and beneficial effects (Franke & Illes, 2006). On the other hand, through the activation of the inhibitory A_1_ adenosine receptor (A_1_R) coupled to G proteins (Dunwiddie & Masino, 2001), adenosine inhibits the release of excitatory neurotransmitters and decreases neuronal excitability, exerting a neuroprotective role (Wardas, 2002).

Recent data have provided evidence for the existence of an association between A_1_R and P2Y_1_R. In a co-transfected cell line model these receptors interact directly (Yoshioka & Nakata, 2004) to generate a hetero-oligomer, which has novel pharmacological and functional characteristics indicating a potential role in the purinergic-signalling cascade. A_1_R and P2Y_1_R have been suggested to be involved in the modulation of brain damage and contribute, alone or in combination, to neuro-degenerative/-regenerative processes (Neary *et al*., 2003; Franke & Illes, 2006; Von Kugelgen, 2006). Moreover, co-localization of these receptors has been demonstrated by immunofluorescence and immunoprecipitation (Yoshioka *et al*., 2002). However, no data are at present available on the precise localization/co-localization of A_1_ and P2Y_1_ receptors at cellular and subcellular levels, or on their reciprocal modulation/functional interaction in the native brain.

Hippocampus is a brain area with a specific vulnerability to injuries, in particular to ischemia (Harry & Lefebvre d'Hellencourt, 2003). In the hippocampus, A_1_R and P2Y_1_R are particularly abundant (Gottlieb & Matute, 1997; Ochiishi *et al*., 1999; Jimenez *et al*., 2000; Moran-Jimenez & Matute, 2000; Zhu & Kimelberg, 2001) and involved in the modulation of glutamate release (Rudolphi *et al*., 1992; Mendoza-Fernandez *et al*., 2000; Masino *et al*., 2002; Koizumi *et al*., 2003; Kawamura *et al*., 2004; Rodrigues *et al*., 2005; Jourdain *et al*., 2007), hence contributing to neurotransmission and neuro-degenerative and -regenerative processes.

In the present work, we investigated the localization/co-localization of A_1_R and P2Y_1_R in rat hippocampus, focusing on glutamatergic synapses and using electron microscopic (EM) quantification ofpostembedding immunogold labelling; this technique allows the precise localization of proteins to be identified at subcellular resolution, on different parts of astrocytes and neurons.

As a first step to investigate the functional A_1_R and P2Y_1_R cross-talk, we studied the A_1_R activation following P2Y_1_R stimulation and vice versa in crude rat hippocampal membranes (HC). For this purpose the [^35^S]guanosine-5′-(γ-thio)-triphosphate ([^35^S]GTPγS) binding assay (Lorenzen *et al*., 1993, 1996) was used. The GTP binding represents the initial step of any GPCR activation and of the intracellular signalling cascade mediated by GPCRs (Lorenzen *et al*., 1996).

## Materials and methods

### Materials

The A_1_R antibody was obtained commercially from Alpha Diagnostic International (San Antonio, TX, USA) and the P2Y_1_R antibody from Alomone Laboratories (Jerusalem, Israel). The data sheets supplied by the manufacturers showed monospecificity by immunoblotting of rat brain. The A_1_R antibody was raised against a 14-amino-acid peptide corresponding to amino acid residues 163–176 of the rat or human receptor protein, i.e. the epitope is in the presumed extracellular N-terminal domain. The P2Y_1_R antibody was raised against a 17-amino-acid peptide (C) RALIYKDLDNSPLRRKS, corresponding to residues 242–258 of rat or human P2Y_1_R, i.e. the epitope location is in the presumed third intracellular loop (i3) between the TM5 and TM6 domains. Secondary antibody, goat anti-rabbit IgG-HRP conjugate, was from Calbiochem (EMD Biosciences, affiliate of Merck kgaA, Darmstadt, Germany). Goat anti-rabbit immunoglobulins coupled to 10-nm or 15-nm gold particles were obtained from Aurion (Wageningen, The Netherlands). Secondary antibody, goat anti-rabbit IgG-HRP conjugate, was from Calbiochem (EMD Biosciences, affiliate of Merck kgaA, Darmstadt, Germany). Electrophoresis reagents were purchased from Bio-Rad (Hercules, CA, USA); full range rainbow molecular weight markers (range 10–250 kDa) were obtained from Amersham Biosciences (Freiburg, Germany).

[^35^S]GTPγS (specific activity 1000 Ci/mmol) was purchased from Amersham Biosciences Europe GmbH (Freiburg, Germany); adenosine deaminase (ADA) was from Roche Diagnostics GmbH (Mannheim, Germany). *N* ^6^-cyclohexyl adenosine (CHA), 8-cyclopentyl-1,3-dipropylxanthine (DPCPX), GDP, guanosine-5′-(γ-thio)triphosphate (GTPγS), 2-methylthio-adenosine 5′-diphosphate (MeSADP), 2′-deoxy-*N* ^6^-methyl adenosine 3′,5′-diphosphate (MRS2179), and protease inhibitors were obtained from Sigma Chemical Co (St Louis, MO, USA). The protein concentration of the samples was established using the protein assay based on the Bradford method from Bio-Rad (Hercules, CA, USA), using bovine serum albumin as a standard. All other reagent grade chemicals were supplied from standard commercial sources.

### Postembedding immunogold cytochemistry

Immunogold electron microscopy quantification was used to study A_1_R and P2Y_1_R receptors in rat hippocampus, focusing on glutamatergic synapses. Animals were treated in accordance with the guidelines of the Norwegian Committees on Animal Experimentation (Norwegian Animal Welfare Act and European Communities Council Directive of 24 November 1986-86/609/EEC). Receptor immunocytochemistry was performed as described previously in Bergersen *et al*. (2005), with some modifications. Wistar rats were deeply anesthetized with an i.p. injection of pentobarbital. Briefly, after transcardiac perfusion with 0.1% glutaraldehyde and 4% formaldehyde in 0.1 m sodium phosphate buffer (NaPi), pH 7.4, the brains from adult male Wistar rats (300 g, *n* = 3) were left *in situ* overnight (4 °C). Then hippocampal specimens were isolated, processed by cryoprotection in different glycerol solutions, snap-frozen in liquid propane cooled by liquid nitrogen and embedded in Lowicryl HM 20 through freeze-substitution. Ultrathin sections (80 nm) were cut with a diamond knife on a Reichert-Jung ultramicrotome and mounted on nickel grids (300 mesh square, Electron Microscopy Sciences, USA). The sections were processed at room temperature in solutions of 0.05 m Tris HCl buffer, pH 7.4 containing 0.3% (w/v, for P2Y_1_R antibody) or 0.1% (for A_1_R antibody) NaCl and 0.1% Triton X-100 (TBST) and completed as stated below.

After ‘etching’ in sodium ethanolate to remove plastic from tissues, sections were incubated in TBST containing 2% human serum albumin (HSA) for 10 min and then overnight (around 20 h) with specific primary antibodies diluted in TBST containing 2% HSA. Antibodies against A_1_R (dilution 2 µg/mL) as well as the antibody against the P2Y_1_R (dilution 4 µg/mL) were used. Sections were then incubated with goat anti-rabbit immunoglobulins coupled to 10 -nm gold particles, diluted 1 : 20 in TBST with 2% HSA and, for A_1_R experiments, with 2 mg/mL polyethylene glycol to suppress the formation of gold particle aggregates.

The ultrathin sections were processed both with single-labelled and double-labelled procedures. In double-labelling experiments (Ottersen *et al*., 1992), sections were treated with the antibody against P2Y_1_ (dilution as above) in the first step (followed by 10-nm gold-labelled secondary antibody) and then with the A_1_R antibody (dilution as above) in the next step (revealed by 15-nm gold-labelled secondary antibody). Exposure to formaldehyde vapours (80 °C, 1 h) was used between the two immunolabelling steps (Wang & Larsson, 1985) to destroy the remaining free anti-IgG binding sites on the first primary and secondary antibodies. Potential cross-reactivity arising from the subsequent use of another secondary antibody that would be directed against the same species is prevented in this manner, allowing the simultaneous detection of two different antigens when using two primary antibodies from the same species, distinguishing the two by means of different gold particle sizes (10 nm for P2Y_1_R, 15 nm for A_1_R). The double-labelling approaches gave similar patterns as the single-labelling protocol.

Negative control experiments also were performed, replacing the incubation with the primary antibodies with 2% HSA in TBST; staining was absent on sections that had been incubated in such a solution. Ultrathin sections were contrasted in uranyl acetate and lead citrate and observed in a Philips CM100 electron microscope.

### Quantitative analysis

**Fig. 1:**
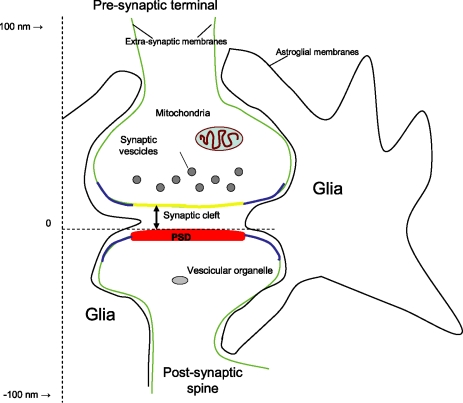
Schematic illustration of a synapse between a presynaptic terminal and a dendritic spine. Analysis of gold particle density (number of gold particles/µm^2^) was performed in specific membrane regions that were defined as presynaptic vesicles membranes, the postsynaptic membrane overlying the postsynaptic density (PSD; red), presynaptic membrane ‘active zone’ (i.e. opposite to the PSD; yellow), pre- and post-perisynaptic membranes on each side of the active zone (blue), extrasynaptic membranes (belonging to either presynaptic terminals or postsynaptic spines/dendrites but excluding the synaptic and perisynaptic membranes; green), postsynaptic intracellular membranes, astroglial plasma membranes (black) and mitochondrial outer membranes (brown). Note that the length of perisynaptic membrane considered corresponds to half the total length of the PSD, on each side (the same is valid for the presynaptic perisynaptic membrane). Figure modified from Bergersen *et al*. (2005).

Electron micrographs were randomly taken in the hippocampus (magnification 34 000×). Gold particles signalling both receptors (P2Y_1_R or A_1_R) were quantified as number of gold particles/µm^2^ at glutamatergic synapses (i.e. asymmetric synapses on dendritic spines) in the stratum radiatum of area CA1 and CA3, as well as in the juxtagranular part of the dentate molecular layer. The former are formed by terminals of ipsilateral (Schaffer collaterals) and commissural axon collaterals from CA3 pyramids), the latter by terminals of mossy cells in the dentate hilus. Only synapses with clearly visible postsynaptic membrane and postsynaptic density were selected for analysis; essentially all synapses with this morphology are glutamatergic in these areas (for identification criteria, see Gylterud Owe *et al*., 2005). Quantitave analyses were carried out on sections single labelled for either P2Y_1_R or A_1_R. Specific membrane compartments were defined and used for quantifications; they correspond to the presynaptic vesicles membranes, the membrane overlying the postsynaptic density (PSD), presynaptic membrane (opposite to the PSD), pre- and post-perisynaptic membranes (corresponding to membrane lateral to the presynaptic active zone and the PSD, extending laterally by half the length of the PSD), extrasynaptic membranes (belonging to either presynaptic terminals or postsynaptic spines/dendrites but excluding the synaptic and perisynaptic membranes), postsynaptic intracellular membranes, astroglia membranes and mitochondrial outer membranes (Fig. 1; cf. Bergersen *et al*., 2005). In addition, gross distribution of gold particles was recorded over the following location categories; presynaptic terminal, postsynaptic spine, synaptic cleft, astrocytes, mitochondria, extracellular and undefined.

Particles located within 40 nm (perpendicular distance between the centre of gold particle and the membrane) from different membranes were recorded. This distance was chosen because 40 nm is approximately equal to the lateral resolution of the immunogold method, i.e. the maximum distance from the centre of the gold particle and the epitope, determined experimentally (Chaudhry *et al*., 1995; Nagelhus *et al*., 1998). The areas sampled were determined by grid point analysis (Gundersen *et al*., 1988; Gundersen *et al*., 1998), and the densities expressed as gold particles/µm^2^. The distance between the centres of gold particles, representing receptors, and the external face of the postsynaptic density was determined along a perpendicular axis. All gold particles located within the postsynaptic spine and the presynaptic terminal were recorded; the width of the synaptic cleft was also measured. The distribution of gold particles was compared with that of grid points placed randomly over the sampled area (Gundersen, V *et al*., 1998; Bergersen *et al*., 2003) in order to relate the distributions of A_1_R and P2Y_1_R to a random distribution.

### Rat brain membrane lysates and Western blotting

Western blot analysis on rat hippocampus and full-brain membranes has been used to confirm the specificity of the antibodies and the presence of the receptors, as a further control.

The whole brain or the hippocampus were removed from Wistar rats (200–300 g; *n* = 3) and immediately processed, keeping on ice. The tissues were suspended in 20 volumes of ice-cold 50 mm Tris HCl, 2 mm MgCl_2_ buffer, pH 7.4, containing EDTA 1 mm and protease inhibitors (benzamidine 0.16 mg/mL, trypsin inhibitor 0.03 mg/mL and bacitracin 0.2 mg/mL) (buffer A). The tissues were then homogenized with a Polytron homogenizer and after centrifugation (48 000 × *g* for 10 min at 4 °C), the membrane pellets were resuspended and re-homogenized in buffer A containing ADA 2 U/mL to obtain a concentration of 50 mg/mL (from original tissue weight). After incubation for 30 min at 37 °C, the samples were centrifuged at 4 °C and each pellet was resuspended to the used concentration, boiled in Laemmli solution for 5 min, centrifuged at room temperature for 5 min and the supernatant used for electrophoresis (or kept at −20 °C until use). Tissue membrane homogenates (50 µg) were then processed by immunoblot following the method previously described (Trincavelli *et al*., 2004) with minor modifications. Briefly, samples were resolved by SDS-PAGE (10%), transferred to nitrocellulose membranes and incubated with primary antibodies against A_1_R (2 µg/mL) (Lasley *et al*., 2000; Ponzio & Hatton, 2005) or P2Y_1_R (4 µg/mL) (Wang *et al*., 2002; Franke *et al*., 2004; Franke *et al*., 2006a) overnight at 4 °C. Blots were developed using the Millipore Immobilon TM Western Chemiluminescent HRP Substrate Reagents (Millipore, MA, USA).

### [^35^S]GTPγS binding assay

For the preparation of rat HC, hippocampus from Wistar rats (200–300 g; *n* = 3) were processed essentially as described by Giuntini *et al*. (2004) and the protein concentration of the samples was determined.

A_1_R coupling to G proteins was evaluated assessing the ability of the selective A_1_R-agonist CHA to stimulate [^35^S]GTPγS binding in membranes pre-treated for 10 min with buffer alone (control HC) or 100 nm MeSADP (MeSADP-treated HC). In parallel, aliquots of control membranes were pre-incubated with the A_1_R selective antagonist DPCPX (50 nm) for 10 min before the CHA-stimulation.

In the same way, P2Y_1_R/G protein coupling was evaluated assessing the ability of the agonist MeSADP to stimulate [^35^S]GTPγS binding in membranes pre-treated for 10 min with buffer alone (control HC) or 100 nm CHA (CHA-treated HC). In parallel, control membranes were also treated with the P2Y_1_R selective antagonist MRS2179 (10 µm), for 10 min before MeSADP-stimulation (MRS2179-treated HC).

Aliquots of membrane homogenate (10 µg) were incubated in 0.1 mL of 25 mm Hepes NaOH, pH 7.4, 5 mm MgCl_2_, 1 mm EDTA, 100 mm NaCl (buffer A) containing ADA (0.2 U/mL), 10 µm GDP and 0.3 nm[^35^S]GTPγS in the presence (stimulated) and absence (basal) of a range of concentrations of CHA or MeSADP (0.1 nm−10 µm). ADA was added to the assay to eliminate the interference of endogenous adenosine in the basal [^35^S]GTPγS binding. Incubation was carried out at 25 °C for 2 h. Unspecific binding was defined in the presence of 100 µm GTPγS; resulted in less than 10% of total binding. Binding reactions were terminated by rapid filtration under vacuum through Whatman GF/C glass fibre filters (Millipore Corporation). The filters were washed three times with 3 mL of 50 mm Tris HCl, 5 mm MgCl_2_, pH 7.4 and then counted in a scintillation cocktail. The concentration-dependent increase in specific [^35^S]GTPγS bindings by agonists was expressed as the per cent increase above the basal unstimulated binding (fixed as 100%). All experiments were performed in duplicate.

### Data analysis

Data from immunogold cytochemistry localization were statistically analysed with one-way anova and/or with Student's *t*-test (two tails, unpaired) by the computer program package GraphPad PRISM Version 4.00 (GraphPad Software, San Diego, CA, USA) and reported as mean ± SEM; statistical significance refers to results where *P* < 0.05 was obtained.

Agonist dose–response curves were analysed by the non-linear regression curve-fitting computer program GraphPad PRISM Version 4.00 and the EC_50_ values were derived. Data are reported as mean ± SEM of four different experiments (performed in duplicate). Statistical analysis (Student's *t*-test, two tails, unpaired) was performed using GraphPad PRISM; significance refers to *P* < 0.05.

## Results

### Immunolocalization

**Fig. 2:**
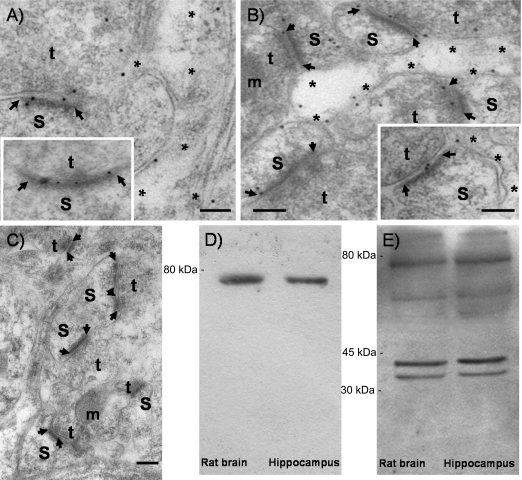
Localization of purinergic receptors in glutamatergic synapses in rat hippocampus by immunogold labelling. Electron micrographs of sections single labelled for A_1_R (A) and for P2Y_1_R (B) are illustrated by examples from CA1 stratum radiatum (A main picture, B inset), CA3 stratum radiatum (A inset) and area dentata (B main picture). Note that both A_1_R and P2Y_1_R were detected on synaptic and glial membranes; both over the postsynaptic density (black arrows) and over the presynaptic (active zone) membranes at glutamatergic synapses and in glia (*). (C) Omission of primary antibody prevented all gold labelling (picture from CA3 stratum radiatum). Scale bar, 100 nm. m, mitochondria; S, postsynaptic dendritic spine; t, presynaptic axon terminal; *, astroglia. (D and E) Western blot analysis of A_1_R (D) and P2Y_1_R (E), in rat whole brain and hippocampus membrane homogenates. Rat tissue membranes were lysed, proteins were separated by 10% polyacrylamide SDS-PAGE and probed with the anti-A_1_R and anti-P2Y_1_R antibodies used for immunocytochemistry.

Postembedding immunogold electron microscopy was used to study A_1_R and P2Y_1_R (Fig. 2A and B) in rat hippocampus, focusing on glutamatergic synapses (i.e. small terminals with asymmetric synapses on dendritic spines) and surroundings glia. For quantitative analysis single-labelled sections, were randomly selected from CA1 and CA3 stratum radiatum, and juxtagranular part of the dentate molecular layer, regions that are particularly high in nerve terminal glutamate and glutamatergic markers (e.g. Cotman *et al*., 1987). As no overt differences were noticed between the areas, they were analysed together. Both A_1_R and P2Y_1_R were detected on synaptic and glial membranes (Fig. 2A and B). Omitting the primary antibodies abolished labelling (Fig. 2C), indicating a low unspecific signal due to the detection system.

A_1_R and P2Y_1_R antibody specificity was addressed further by Western blot analysis, using rat whole brain and hippocampus membrane fractions (Fig. 2D and E). Results confirmed the presence of the two receptors and their antibody specificity. The A_1_R immunoreactive band corresponds to the dimer form of A_1_Rs (79 kDa; Fig. 2D) (Ciruela *et al*., 1995; Blum *et al*., 2002), while the P2Y_1_R multiline pattern shows immunoreactive bands at 76, 40 and 35 kDa, corresponding to dimeric and monomeric variants (Fig. 2E), in agreement with the literature data (Hoffmann *et al*., 1999; Waldo & Harden, 2004).

**Fig. 3:**
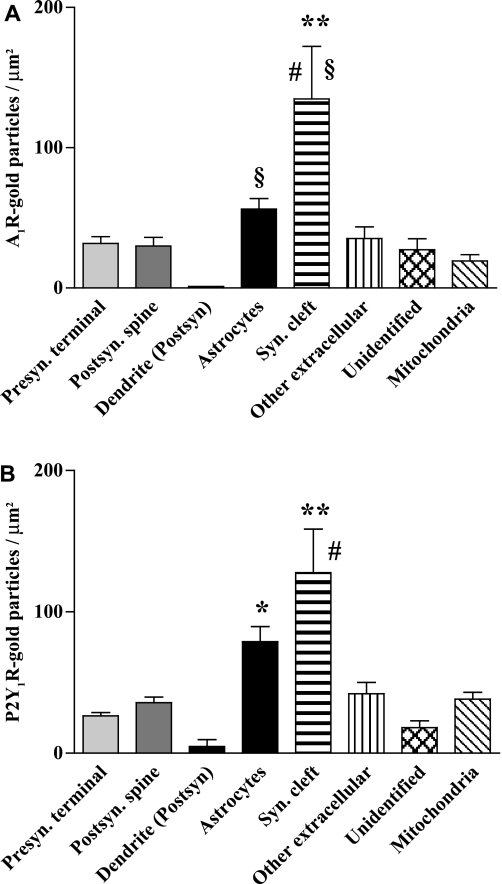
Distribution of immunogold particles indicating A_1_R (A) or P2Y_1_R (B) in different intra- and extracellular compartments. Note that both receptors are enriched at synapses and astroglia, compared to all other compartments. Data, from single labelling experiments, report the localization of receptors as the density (gold particles/µm^2^; mean ± SEM) in the different compartments in the vicinity of hippocampal glutamatergic synapses (i.e. asymmetric synapses on spines, see Materials and methods; randomly selected, from *n* = 3 animals; five grids analysed for A_1_R and six grids for P2Y_1_R); the areas of the compartments were measured by point analysis (see Materials and methods). The numbers of gold particles on each structure are, in order 323, 230, 1, 52, 24, 31, 15 for A_1_R; 330, 311, 5, 112, 35, 31, 32 for P2Y_1_R. Areas analysed for each compartment are, in order 10.8, 7.7, 0.1, 1.1, 0.2, 0.6, 1.3, 1.0 µm^2^ for A_1_R, 12.1, 8.8, 0.2, 2.1, 0.2, 0.9, 1.7, 0.8 µm^2^ for P2Y_1_R. (A) A_1_R. **P < 0.01, one-way anova, all compartments compared to mitochondria (Dunnett's multiple comparison test). Synaptic cleft is significantly different compared to all other domains (^#^*P* < 0.01, one-way anova, Tukey's multiple comparison test). Astrocytes and synaptic cleft columns showed higher receptor density (^§^*P* < 0.05, Student's *t*-test, two-tails, unpaired) compared to mitochondria. (B) P2Y_1_R; *P < 0.05, **P < 0.01 all domains compared to mitochondria, one-way anova (Dunnett's multiple comparison test). Synaptic cleft is significantly different compared to all other domains (^#^*P* < 0.001 vs. all except astrocytes, *P* < 0.01 vs. astrocytes, One-way anova, Tukey's multiple comparison test).

The receptor distribution and localization was quantified in different subcellular structures (Fig. 3A and B). The localization of both receptors was expressed as the area density, the number of gold particles/µm^2^, in different structures in the vicinity of hippocampal glutamatergic synapses, determining the area by point analysis (Gundersen *et al*., 1988). Assuming the mitochondria do not contain the receptors, the density of gold particles over mitochondria may be taken to represent unspecific background binding of antibodies. (This may be an overestimate of background labelling, as the protein concentration in mitochondria is exceptionally high.) Both A_1_R and P2Y_1_R were highly concentrated in the synaptic cleft area and in adjacent astrocytes, which were the only compartments significantly higher than mitochondria (Fig. 3A and B). Because the spatial resolution of our immunolabelling method is of the same order of magnitude as the distance between different compartments/membranes in the tissue, an individual gold-particle cannot be directly ascribed to any single structure. Therefore, the association of gold particles with membranes was analysed in several different ways.

**Fig. 4:**
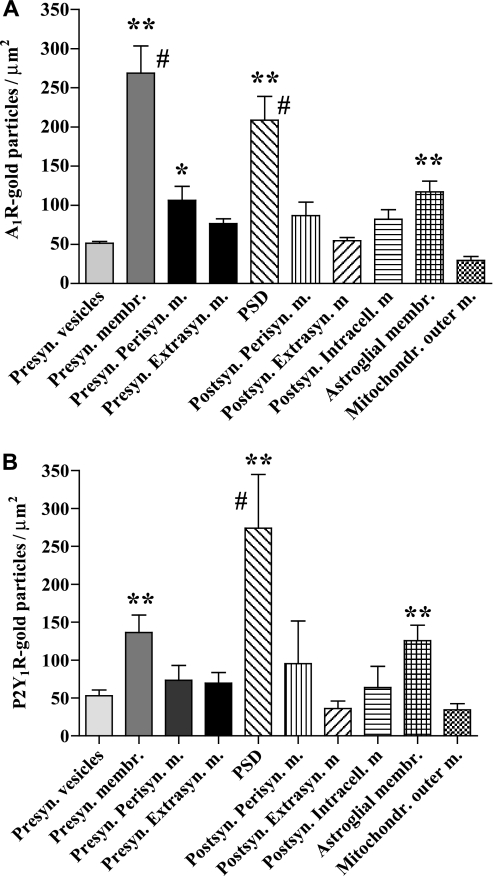
Distribution of immunogold particles indicating A_1_R (A) or P2Y_1_R (B) over different categories of cellular membranes in the vicinity of hippocampal synapses. Both receptor types are enriched over presynaptic as well as postsynaptic membranes and over astroglial membranes. Only particles located within a distance equal to 10 nm from each kind of membrane were recorded, to minimize ‘cross firing’ effects from immunoreactivity in nearby structures. The data are presented in the same way, and are from the environments of the same synapses, as are the data in Fig. 3. The sizes of the areas sampled for gold particles (± 10 nm on each side of the membrane) were determined by point analysis. Areas analysed for each category are, in order 3.8, 0.5, 0.3, 1.4, 0.7, 0.3, 1.1, 0.3, 0.6, 0.6 µm^2^ for A_1_R; 5.1, 0.8, 0.3, 1.6, 0.8, 0.4, 2.0, 0.6, 1.2, 0.7 µm^2^ for P2Y_1_R. The numbers of gold particles within 10 nm from each membrane column are, in order 196, 126, 22, 98, 141, 19, 54, 32, 57, 12 for A_1_R.; 260, 81, 15, 101, 124, 26, 74, 36, 116, 27 for P2Y_1_R. The density of gold particles over the outer membrane of mitochondria gives an estimate of the background level. (A) *P < 0.05, **P < 0.01, all columns compared to mitochondria background level, one-way anova (Dunnett's multiple comparison test). Presynaptic membrane and PSD are significantly different compared to all other membrane categories (^#^*P* < 0.01, one-way anova, Tukey's multiple comparison test). (B) **P < 0.01, all columns compared to mitochondria background level, one-way anova (Dunnett's multiple comparison test). PSD is significantly different compared to all other columns except for presynaptic membranes and astroglial membranes (^#^*P* < 0.01, one-way anova, Tukey's multiple comparison test).

First, we investigated the distribution of gold particles associated with each kind of cellular membrane, in the vicinity of hippocampal synapses, recording only particles within a distance equal to 10 nm from each kind of membrane to minimize ‘cross firing’ effects from immunoreactivity in nearby structures. Immunogold single-labelling of hippocampal sections showed that A_1_R (Fig. 4A) as well as P2Y_1_R (Fig. 4B) are mainly associated with presynaptic membranes, postsynaptic membranes overlying the PSDs, and astroglial membranes. The high A_1_R presence on presynaptic-perisynaptic membranes (*P* < 0.05, one-way anova, value vs. mitochondria) could be influenced from receptors that are located in the active zone or as ‘reserve’ (Rebola *et al*., 2003); anyway the values on perisynaptic and extrasynaptic membranes, both pre- and post-, can be influenced from receptors that are instead on astroglia membranes, for the tight contact between these and neurons. No other columns differed from the background level, as indicated by the density of gold particles over mitochondrial outer membranes (*P* > 0.05, one-way anova). Further, values at the presynaptic membrane and at the membrane covering the PSD were significantly different compared to all other locations, confirming that A_1_Rs are associated with these membranes.

**Fig. 5:**
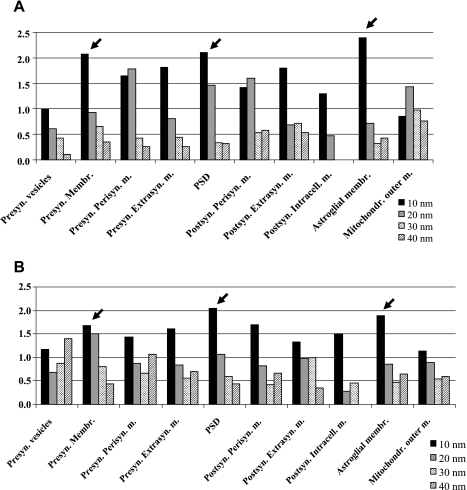
Histogram of gold particles/random points ratio; gold particles were grouped in bins depending the distance (10, 20, 30, 40 nm) from each kind of membrane, at hippocampal glutamatergic synapses and surrounding glia. The percentage of the total number of gold particles that occurred in each bin was divided by the percentage of the total random points (within 40-nm distance) that occurred in the same bin; the ratio gives the distribution of receptors relative to a random distribution. A ratio > 1 for the shorter distance bins (10 nm and 20 nm), indicates a close association with the membrane observed. Note the higher ratios (arrows) for short distances at presynaptic active zone membranes, postsynaptic membranes overlying the PSD and astroglial membranes, for both A_1_R (A) and P2Y_1_R (B)

We then compared the distribution of gold particles representing A_1_R and P2Y_1_R with the distribution of points spread randomly over the pictures (Fig. 5). The gold particles/random points ratio was determined at different distances, from different membrane domains, sorted into bins (10-20-30-40 nm). For the shortest distance (10 nm) bins, the highest ratio values (approximately 2) were obtained, for both A_1_R and P2Y_1_R, at presynaptic active zone membranes, postsynaptic membranes overlying the PSD and astroglial membranes (Fig. 5A and B), i.e. the same membrane domains that show the highest labelling according to the analysis of Fig. 4. Also several other neuronal membranes, but not mitochondrial outer membranes, had ratios higher than one, consistent with a moderate enrichment of receptors in them. As previously noted, the densities in perisynaptic and extrasynaptic membranes could be influenced from gold particles in active zone or in astroglial membranes.

**Fig. 6:**
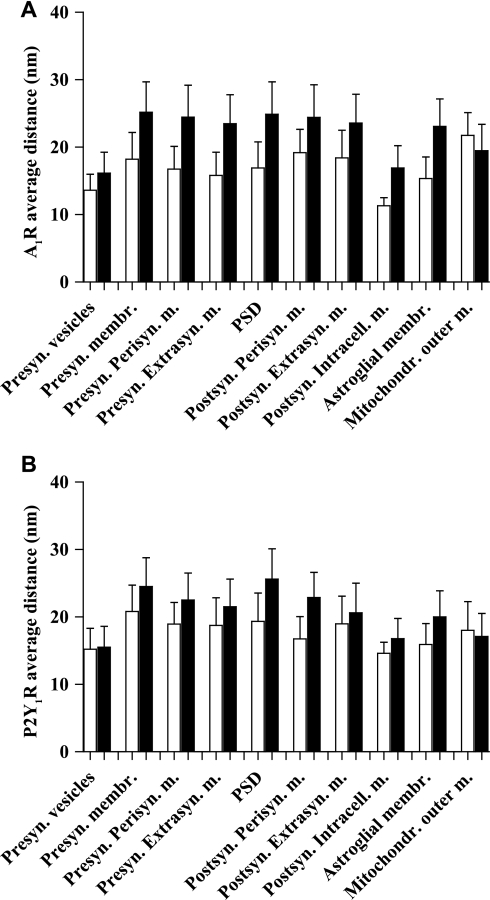
Average distances of immunogold particles from membranes. The values are the mean ± SEM of the distances of gold particles (white bars) or random points (black bars) located within a distance equal to 40 nm from different membrane categories, in the vicinity of glutamatergic synapses in rat hippocampus. Note that for both A_1_R (A) and P2Y_1_R (B) the mean distance of gold particles is shorter than that of random points in all membranes, except in mitochondria outer membranes (which represent background labelling). The total number of particles counted is for A_1_R gold particles 286, 241, 34, 159, 230, 38, 101, 37, 91, 241; for P2Y_1_R gold particles 392, 238, 41, 183, 247, 47, 145, 44, 207, 44; for A_1_ random points 457, 212, 46, 313, 218, 48, 187, 39, 149, 177; for P2Y_1_ random points 457, 127, 47, 270, 132, 55, 269, 60, 162, 63.

The average distance of immunogold particles from membranes was then measured and compared with a random distribution (Fig. 6). Note that for both A_1_R (Fig. 6A) and P2Y_1_R (Fig. 6B) the mean distance of gold particles is shorter than that of random points in all membranes, except in mitochondrial outer membranes (which represent an estimate of background labelling).

**Fig. 7:**
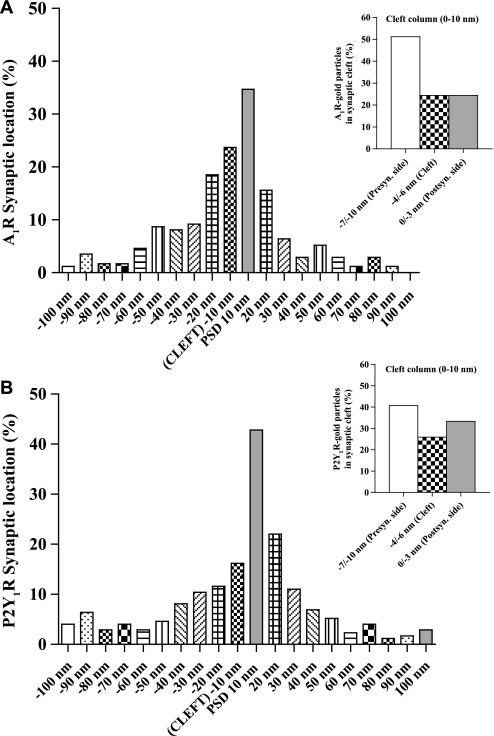
Distribution of immunogold particles across the synaptic cleft. The histograms on the left show the percentage of gold particles representing A_1_R (A) and P2Y_1_R (B) in the synaptic area, as a function of the distance up to 100 nm from the postsynaptic membrane at the PSD. The distance between the centre of gold particles and the external face of the PSD was determined along a perpendicular axis (cf. Fig. 1). Positive columns (10 to 100 nm) represent the postsynaptic spine; negative columns (−20 to −100 nm) the presynaptic terminal; the −10 nm column represents the synaptic cleft (i.e. position 0 to −10 nm). The actual width of the synaptic cleft was 11.5 ± 0.4 nm (average ± SEM, *n* = 79). Numbers of gold particles considered in the analysis are *n* = 266 for A_1_R; *n* = 296 for P2Y_1_R. All synapses previously analysed have been processed in this analysis. Note that the highest particle counts for A_1_R as well as P2Y_1_R were in the PSD bin. The histograms on the right show the distribution of immunogold particles within the synaptic cleft. The percentage of the total number of gold particles located in the synaptic cleft (*n* = 41 for A_1_R; *n* = 28 for P2Y_1_R), in function of the distance from the outer face of the PSD; presynaptic side, position between 0 and −3 nm; postsynaptic side, between −6 and −10 nm; middle of cleft, between −3 and −6 nm. The distributions within the synaptic cleft indicate that both A_1_R (A) and P2Y_1_R (B) are located presynaptically as well as postsynaptically.

To dissect further the synaptic localization of A_1_R and P2Y_1_R, all the hippocampal glutamatergic synapses previously analysed were processed to record the percentage distribution of both receptors as a function of the perpendicular distance from the postsynaptic membrane overlying the PSD (Fig. 7). The percentage distribution of A_1_Rs in the synaptic area (Fig. 7A, left) showed that A_1_R are located over both the PSD and the presynaptic membrane, although the density is highest over the latter. The influence of the extracellular location of the epitope recognized by the A_1_R antibody may have contributed to the high level of gold particles located in the synaptic cleft. A_1_R-gold particles situated over the synaptic cleft were preferentially associated with the presynaptic membrane (Fig. 7A, right), indicating a shared distribution of the receptor between the presynaptic and postsynaptic sides. P2Y_1_Rs were relatively more distributed towards the PSD membrane (Fig. 7B, left), compared to A_1_Rs, and gold particles in the cleft showed less enrichment on the presynaptic side (Fig. 7B, right). These results are consistent with a high degree of co-localization of the two types of receptor, but with relatively less of P2Y_1_R than of A_1_R on the presynaptic side.

**Fig. 8:**
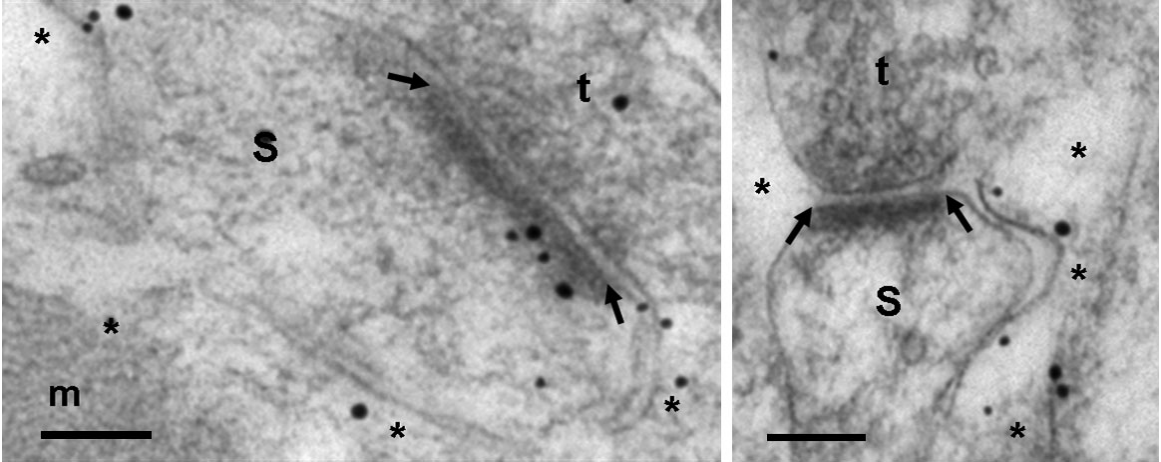
Qualitative co-localization of P2Y_1_R and A_1_R in glutamatergic synapses (randomly selected) and surrounding glia, in rat hippocampus CA3 stratum radiatum. Electron micrograph of a section double labelled for A_1_R (large gold particles, 15 nm) and P2Y_1_R (small gold particles, 10 nm). The double-labelling approach gave similar patterns as the single-labelling protocol. Note that the receptors were detected together in the same structures. A_1_R and P2Y_1_R appeared co-localized (qualitative data) on the PSD (black arrows) and on astroglia (*). Scale bar, 100 nm. M, mitochondria; S, postsynaptic spine; t, presynaptic terminal; *, astroglia.

In conclusion, A_1_R and P2Y_1_R are co-localized in synaptic and astroglial membranes in glutamatergic synapses and surrounding glial membranes in the hippocampus. The quantitative analyses of single-labelling data were confirmed by the qualitative analysis of double-labelling experiments (Fig. 8). The double-labelling approach gave similar patterns as the single labelling protocol; the receptors were detected together in the same structures and both were associated with synaptic and astroglial membranes (Fig. 8).

### Functional assays

**Fig. 9:**
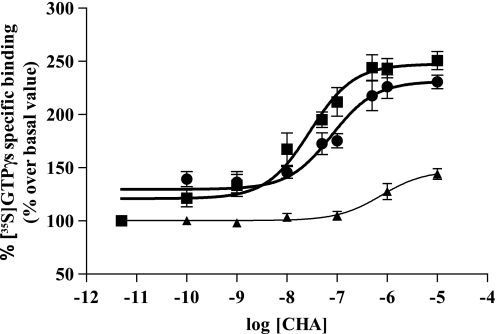
Effect of P2Y_1_R activation on A_1_R agonist-mediated G protein coupling. Dose–response curves of CHA-stimulated [^35^S]GTPγS binding were obtained incubating control HC (▪) and MeSADP-treated (100 nm) HC (•) with different agonist concentration (0.1 nm−10 µm). Data are expressed as percentage of [^35^S]GTPγS specific binding over basal value, set to 100%, and are reported as mean ± SEM (*n* = 4), all performed in duplicate. Student's *t*-test, two-tails, unpaired: *P* = 0.004 for EC_50_ of Control HC vs. MeSADP-treated HC. Aliquots of HC were also pre-exposed to the selective A_1_R antagonist DPCPX (50 nm; ▴) and then stimulated by CHA (0.1 nm−10 µm).

The effect of P2Y_1_R activation on A_1_R/G protein coupling was measured in HC by evaluating the ability of the A_1_R agonist CHA to stimulate G protein activation following 100 nm MeSADP pre-incubation (for 10 min). In control HC, CHA stimulated G protein activation with an EC_50_ of 30.60 ± 3.99 nm(Fig. 9). To test the CHA-mediated selective A_1_R activation in our model, we also stimulated HC in the presence of the selective A_1_R antagonist DPCPX (50 nm; pre-incubated for 10 min). DPCPX prevented CHA-mediated G protein coupling in HC (Fig. 9), showing that the G protein activation is selectively driven by A_1_R. The CHA-mediated effect was abolished in the presence of the selective A_1_R antagonist DPCPX (50 nm), confirming A_1_R specific activation.

Membrane pre-incubation with 100 nm MeSADP induced a right-shift of the CHA dose/response curve (EC_50_ = 106.26 ± 16.39 nm), suggesting a significant impairment in A_1_R-G protein coupling when P2Y_1_ receptor is activated (*t*-test, two-tails, unpaired *P* = 0.004 for EC_50_ of MeSADP-treated HC vs. control HC; Fig. 9).

**Fig. 10:**
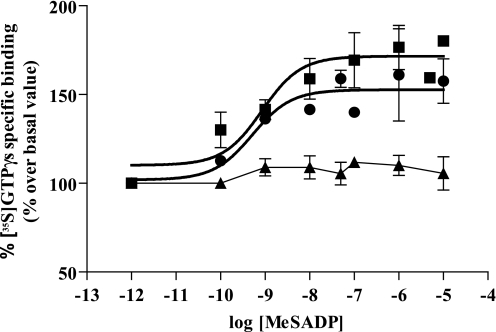
Effect of A_1_R activation on P2Y_1_R agonist-mediated G protein coupling. Dose–response curves of MeSADP-stimulated [^35^S]GTPγS binding were obtained incubating control HC (▪) and CHA-treated (100 nm) HC (•) with different agonist concentration (0.1 nm−10 µm). Data are expressed as percentage of [^35^S]GTPγS specific binding over basal value set to 100% and are reported as mean ± SEM (*n* = 4), all performed in duplicate. Student's *t*-test, two-tails, unpaired: *P* = 0.03 for EC_50_ of control HC vs. CHA-treated HC. Aliquots of HC were also pre-exposed to the selective P2Y_1_R antagonist MRS2179 (10 µm; MRS2179-treated HC; ▴) and then stimulated by MeSADP (0.1 nm−10 µm). Data are expressed as percentage of [^35^S]GTPγS specific binding over basal value set to 100% and are reported as mean ± SEM (*n* = 4), all performed in duplicate.

On the other hand, the A_1_R activation effect on P2Y_1_R/G protein coupling was measured by evaluating the ability of the P2Y_1_R agonist MeSADP to stimulate G protein activation in the absence or the presence of 100 nm CHA (pre-incubated for 10 min). In control HC, MeSADP stimulated [^35^S]GTPγS binding with an EC_50_ of 0.851 ± 0.147 nm(Fig. 10).

To test the selectivity of MeSADP for P2Y_1_ receptor subtype in our model, we also stimulated HC in the presence of the selective P2Y_1_R antagonist 10 µm MRS2179 (pre-incubated for 10 min). MRS2179 blocked MeSADP-mediated G protein coupling in HC (Fig. 10), showing this G protein activation is selectively driven by P2Y_1_R, and other P2Y receptors sensitive to MeSADP (P2Y_12−13_) have no significant signal component in rat hippocampus. Membrane CHA pre-incubation induced a significant increase in MeSADP potency to activate P2Y_1_R-G protein coupling, with an EC_50_ of 0.393 ± 0.058 nm (*t*-test; two-tails, unpaired *P* = 0.03 vs. control HC), without significantly affecting the G protein coupling efficacy level of the agonist dose/response curve (Fig. 10).

Cell treatment with MeSADP or CHA induced an increase in the basal [^35^S]GTPγS binding value, thus suggesting the agonists affect the G protein activation state. Values are reported as fmol/mg of proteins; control HC = 183.96 ± 30.2; MeSADP HC = 298.65 ± 30.18; CHA HC = 317.93 ± 8.42 (*t*-test, two-tails, unpaired, *P* = 0.047 for MeSADP-treated HC vs. control HC; *P* = 0.017 for CHA-treated HC vs. control HC).

## Discussion

### Localization and co-localization

This is the first study on A_1_R and P2Y_1_R localization and co-localization in hippocampus by postembedding immunogold electron microscopy analysis. This high resolution technique is able to show the precise subcellular localization of receptors in different subcellular compartments and cell populations (e.g. Bergersen *et al*., 2003). The purinergic receptors proved to be mainly associated with membrane domains. Single-labelling immunolocalization data showed a significant enrichment of both A_1_R and P2Y_1_R mainly in postsynaptic membranes at the PSD, in presynaptic active zones, and in astroglial membranes at glutamatergic synapses and surrounding glia in the rat hippocampus. The same conclusion was arrived at by analysing the data in different ways, in order to partly overcome the limits posed by ‘cross firing’ effects antigen located in closely spaced neighbouring membranes. Because of these, the exact labelling densities of the individual membranes cannot be determined. While the three membrane categories mentioned contain higher levels of both of the purinergic receptors studied, low or moderate labelling may exist in other membrane categories. The data suggest that there may be relatively higher densities of A_1_R than of P2Y_1_R at the presynaptic compared to the postsynaptic membrane. Part of this observed difference might be attributable to the fact that the antibodies were to extracellular and intracellular epitopes, respectively, although the inner and outer surfaces of the plasma membrane are only 4–5 nm apart, i.e. an order of magnitude less than the lateral resolution of the immunogold method. However, the main conclusion is that the two receptor types are similarly distributed, compatible with a high degree of co-localization. This was born out by double-labelling experiments that showed A_1_R and P2Y_1_R to be closely spaced along synaptic and glial membranes.

In parallel, the specificities of A_1_R and P2Y_1_R antibodies used in immunohistochemistry, were assessed by immunoblotting assay using rat whole brain and hippocampus membrane fractions. The obtained results were according to literature data (Hoffmann *et al*., 1999; Blum *et al*., 2002; Moore *et al*., 2002; Waldo & Harden, 2004) and confirmed that antibodies selectively recognized A_1_ and P2Y_1_ receptors in hippocampal membranes.

Our high resolution data serve to extend and reconcile previous reports obtained with lower resolution methods. Thus A_1_R immunoreactivity has been reported in hippocampus both at presynaptic and postsynaptic terminals but not at glial cells (Ochiishi *et al*., 1999); an immunohistochemical study in the rat hippocampus has concluded that A_1_Rs were mostly located in axons rather than in nerve terminals(Swanson *et al*., 1995), whereas work on synaptosomal fractions (Rebola *et al*., 2003) has suggested that A_1_Rs are enriched in nerve terminals and are mainly located in synapses, both in the presynaptic active zone and in the PSD membranes.P2Y_1_R immunoreactivity has been found in astroglia and in different kinds of neurons in hippocampus (Moran-Jimenez & Matute, 2000), especially in ischemic sensitive areas while at the same time another study reported a striking neuronal localization for P2Y_1_R (human brain, Moore *et al*., 2000). P2Y_1_Rs have been reported to be present and active on astrocytes all around the brain (Franke *et al*., 2001; Volonté*et al*., 2006). A high degree of co-localization of A_1_R and P2Y_1_R has been found in rat hippocampus by immunofluorescence experiments but without cellular and subcellular identification (Yoshioka *et al*., 2002).

We studied A_1_Rs and P2Y_1_Rs in the hippocampal region considering the fact that the hippocampus has been identified as a major target site for numerous disease processes (Bachevalier & Meunier, 1996; Harry & Lefebvre d'Hellencourt, 2003), and considering the generally assumed involvement of purinergic receptors in patho-physiological mechanisms and in the modulation of brain damage (Fredholm, 1997; Franke *et al*., 2006b). Ischemia, to which hippocampus is particularly vulnerable, produces a marked increase in glutamate within the brain extracellular space (Benvensiste *et al*., 1984; Hagberg *et al*., 1985), thereby triggering excitotoxic injuries (Choi & Rothman, 1990). Because of the importance of glutamate in pathological conditions and because its release, in neurons and in astrocytes, is modulated both through A_1_R (Rudolphi *et al*., 1992; Masino *et al*., 2002) and P2Y_1_R (Rodrigues *et al*., 2005; Franke *et al*., 2006a; Jourdain *et al*., 2007), the present study was focused on A_1_R–P2Y_1_R localization and co-localization within and in the vicinity of glutamatergic synapses.

Our results provide direct morphological support for the previous suggestions that both of these receptors contribute to and interact in the modulation of glutamate release (Rudolphi *et al*., 1992; Mendoza-Fernandez *et al*., 2000; Masino *et al*., 2002; Kawamura *et al*., 2004; Rodrigues *et al*., 2005).

### Functional interaction

The functional interaction of A_1_Rs and P2Y_1_Rs suggested by the morphological observations was subsequently confirmed through measurement of G protein activation initiated by the A_1_R agonist, CHA, or the P2Y_1_R agonist, MeSADP, respectively, and modification of the response through pre-incubation with the other agonist. Because the receptors on study are coupled to different G protein subtypes (Munshi *et al*., 1991; Yoshioka & Nakata, 2004) and to different intracellular signalling pathways, the [^35^S]GTPγS binding method was chosen to investigate the A_1_R–P2Y_1_R interaction and their reciprocal modulation at the membrane level, allowing any change in their functioning to be determined independently of the second messenger systems activated (Lorenzen *et al*., 1996).

According to literature data (Gao *et al*., 2003; Dixon *et al*., 2004; Niebauer *et al*., 2005), the selected agonist pre-incubation times and concentrations pre-stimulating A_1_R and P2Y_1_R (100 nm CHA and 100 nm MeSADP, respectively) allow a selective and maximal receptor activation.

The EC_50_ for CHA in stimulating A_1_R-G protein coupling was around 30 nm. Although CHA has been reported to block [^3^H]DPCPX binding at A_1_R with a *K*_i_ of approximately 5 nm in rat hippocampus (Maemoto *et al*., 1997), according to our data, higher EC_50_ values for different A_1_R agonists in the [^35^S]GTPγS binding assay have been found (Lorenzen *et al*., 1996; Cordeaux *et al*., 2004). In order to obtain a selective stimulation of A_1_R, without interference of other receptor subtypes, we have chosen treatment of hippocampal membranes with 100 nm agonist CHA. In fact in hippocampus, although A_1_Rs are the mainly expressed adenosine receptors, the presence of low amounts of A_2A_ and A_3_ receptors have been described (Duarte *et al*., 2006; Lopes *et al*., 2003). Nevertheless, these receptors have a different agonist pharmacological profile; CHA, in fact, shows a high affinity towards A_1_Rs (Murphy & Snyder, 1982; Maemoto *et al*., 1997) and a low affinity towards A_2A_ ones (Cunha *et al*., 1996; Gao *et al*., 2003). Even if, in our experimental conditions, the EC_50_ for CHA in stimulating A_1_R-G-protein coupling was around 30 nm, the real binding potency of CHA to A_1_R sites is around a few nanomolar units or indeed in the subnanomolar range in rat hippocampus, so the agonist at 100 nm is able to selectively saturate A_1_R binding sites. This was confirmed by an antagonist dose–response curve, DPCPX was able to completely abrogate the effects mediated by CHA from 1 to 100 nm. At higher agonist concentrations the antagonistic effect of DPCPX was reduced suggesting that the agonist binds to a different receptor population, probably identifiable with A_2A_ receptor sites.

On the other hand, in our results, MeSADP showed subnanomolar potency in stimulating P2Y_1_R. Because of the absolute potency of nucleoside tri- and diphosphates for P2Y receptors is dependent on the levels of receptor protein expression, typical EC_50_ values are not easily defined for specific agonists at particular P2Y receptor subtypes in different tissue preparations and cell lines (Volontè*et al*., 2006). In accordance with our results, low nanomolar and subnanomolar EC_50_ values have been reported for MeSADP towards human P2Y_1_R, expressed in astrocytoma 1321 N1 cells (Palmer *et al*., 1998; Niebauer *et al*., 2005), and rat P2Y_1_R, expressed in HEK 293 cells (Vohringer *et al*., 2000).

MeSADP is the principal agonist not only at P2Y_1_R but also at P2Y_12−13_ receptors, that are coupled to G_I_ proteins and are expressed (mRNA) in the brain (Hollopeter *et al*., 2001; Zhang *et al*., 2002; Sasaki *et al*., 2003), even if not at high level in the hippocampus (Fumagalli *et al*., 2004; Amadio *et al*., 2006). To confirm that in our model the MeSADP-mediated G-protein activation was mainly driven by P2Y_1_R, we also stimulated hippocampal membranes in the presence of the selective P2Y_1_R antagonist, MRS2179. MRS2179 was able to block the MeSADP-mediated response, confirming the P2Y_1_R involvement as no antagonistic effects have been demonstrated on P2Y_12−13_ receptors at the MRS2179 concentrations used (Moro *et al*., 1998; Von Kugelgen, 2006).

The results obtained on A_1_R–P2Y_1_R cross-talk in hippocampus showed that, stimulating one receptor, the functioning of the other was changed. In particular, P2Y_1_R pre-activation caused an impairment in A_1_R–G protein coupling with a reduction in A_1_R agonist potency; on the other hand, A_1_R pre-activation induced an increase in P2Y_1_R functional coupling to G proteins. Our results are in agreement with the previously reported reduction in the A_1_R ligand affinities in cells co-expressing both A_1_R and P2Y_1_R (Yoshioka *et al*., 2001). Various studies have reported that ATP, massively released after brain damage, acts to modulate not only its own P2Y_1_R but also A_1_R (Hourani *et al*., 1991; Piper & Hollingsworth, 1996; Masino *et al*., 2002; Fredholm *et al*., 2003; Yoshioka & Nakata, 2004).

The functional consequence of this A_1_–P2Y_1_ receptor cross-talk is complicated by the availability time and the balance of their endogenous ligands. Extracellular ATP, rapidly available due to direct release into the extracellular space, and adenosine, available after ATP breakdown, are tightly regulated by rapid metabolism and re-uptake (Zimmermann, 2000) and can be differently regulated in physiological or pathological conditions; in fact the ecto-nucleotidase chain has proved to be up-regulated in ischemically damaged tissues (Braun *et al*., 1998).

Data, at present, have shown the A_1_R–P2Y_1_R interaction mechanism may be used to fine-tuning the purinergic signalling, including the inhibition of neurotransmission (Nakata *et al*., 2004). Considering the new information available and the A_1_R and P2Y_1_R involvement in glutamatergic transmission modulation (Mendoza-Fernandez *et al*., 2000; Masino *et al*., 2002; Kawamura *et al*., 2004; Rodrigues *et al*., 2005), we can speculate that there is an A_1_R–P2Y_1_R cross-talk in rat hippocampal glutamatergic synapses and surroundings glia, where these receptors are co-localized. This might therefore be one of the mechanisms for the adenine nucleotide-mediated inhibition of glutamatergic neurotransmitter release. Therefore, as suggested for adenosine A_1_ and A_2A_ receptors in striatal (Ciruela *et al*., 2006) and hippocampal (Rebola *et al*., 2005) glutamatergic nerve terminals, a cross-talk/heteromerization of A_1_R–P2Y_1_R could exert a fine-tuning modulation of glutamatergic neurotransmission, providing a switch mechanism by which low and high concentrations of adenosine or purines could regulate glutamate release.

Because of the high level of complexity of purinergic receptor signalling (Volontè*et al*., 2006) and the regulation of glia–neuron and glia–glia communications by extracellular purines (Franke *et al*., 2006b; Jourdain *et al*., 2007), the present work opens the way to further investigation of the A_1_R–P2Y_1_R system interaction on astrocyte cell populations, which communicate bi-directionally with neurons (Newman, 2003; Bezzi *et al*., 2004; Jourdain *et al*., 2007) and contribute to damage or to regeneration after CNS injury (Franke *et al*., 2001; Anderson *et al*., 2003).

## Abbreviations

A_1_R: adenosine receptor A_1_
ADA: adenosine deaminase
CHA: *N*^6^-cyclohexyladenosine
DPCPX: 8-cyclopentyl-1,3-dipropylxanthine
GPCR: G protein coupled receptors
GTPγS: guanosine-5′-(γ-thio)triphosphate
HSA: human serum albumin
HC: hippocampal membranes
MeSADP: 2-methylthio-adenosine 5′-diphosphate
MRS2179: 2′-deoxy-*N*^6^-methyl adenosine 3′,5′-diphosphate
P2Y_1_R: purinergic receptor P2Y_1_
PSD: postsynaptic density
TBST: Tris-buffered saline with Triton X-100

## References

[b1] Amadio S, Tramini G, Martorana A, Viscomi MT, Sancesario G, Bernardi G, Volonte C (2006). Oligodendrocytes express P2Y_12_ metabotropic receptor in adult rat brain. Neuroscience.

[b2] Anderson MF, Blomstrand F, Blomstrand C, Eriksson PS, Nilsson M (2003). Astrocytes and stroke: networking for survival?. Neurochem. Res.

[b3] Bachevalier J, Meunier M (1996). Cerebral ischemia: are the memory deficits associated with hippocampal cell loss?. Hippocampus.

[b4] Benvensiste H, Drejer J, Schousboe A, Diemer NH (1984). Elevation of the extracellular concentrations of glutamate and aspartate in rat hippocampus during transient cerebral ischemia monitored by intracerebral microdialysis. J. Neurochem.

[b5] Bergersen LH, Magistretti PJ, Pellerin L (2005). Selective postsynaptic co-localization of MCT2 with AMPA Receptor GluR2/3 subunits at excitatory synapses exhibiting AMPA receptor trafficking. CerebCortex.

[b6] Bergersen L, Ruiz A, Bjaalie JG, Kullmann DM, Gundersen V (2003). GABA and GABAa receptors at hippocampal mossy fibre synapses. Eur. J. Neurosci.

[b7] Bezzi P, Gundersen V, Galbete JL, Seifert G, Steinhauser C, Pilati E, Volterra A (2004). Astrocytes contain a vesicular compartment that is competent for regulated exocytosis of glutamate. Nature Neurosci.

[b8] Blum D, Gall D, Galas MC, d'Alcantara P, Bantubungi K, Schiffmann NS (2002). The adenosine A1 receptor agonist adenosine amine congener exerts a neuroprotective effect against the development of striatal lesions and motor impairments in the 3-nitropropionic acid model of neurotoxicity. J. Neurosci.

[b9] Braun N, Zhu Y, Krieglstein J, Culmsee C, Zimmermann H (1998). Upregulation of the enzyme chain hydrolyzing extracellular ATP after transient forebrain ischemia in the rat. J. Neurosci.

[b10] Burnstock G (2004). Introduction: P2 receptors. Curr. Top. Med. Chem.

[b11] Chaudhry FA, Lehre KP, Van Lookeren Campagne M, Ottersen OP, Danbolt NC, Storm-Mathisen J (1995). Glutamate transporters in glial plasma membranes: highly differentiated localizations revealed by quantitative ultrastructural immunocytochemistry. Neuron.

[b12] Choi DW, Rothman SM (1990). The role of glutamate neurotoxicity in hypoxic-ischemic neuronal death. Annu. Rev. Neurosci.

[b13] Ciruela F, Casado V, Mallol J, Canela EI, Lluis C, Franco R (1995). Immunological identification of A1 adenosine receptors in brain cortex. J. Neurosci. Res.

[b14] Ciruela F, Casado V, Rodrigues RJ, Lujan R, Burgueno J, Canals M, Borycz J, Rebola N, Goldberg SR, Mallol J, Cortes A, Canela EI, Lopez-Gimenez JF, Milligan G, Lluis C, Cunha RA, Ferre S, Franco R (2006). Presynaptic control of striatal glutamatergic neurotransmission by adenosine A1–A2A receptor heteromers. J. Neurosci.

[b15] Cordeaux Y, Ijzerman AP, Hill JS (2004). Coupling of the human A1 adenosine receptor to different heterotrimeric G proteins: evidence for agonist-specific G protein activation. Br. J. Pharmacol.

[b16] Cotman CW, Monaghan DT, Ottersen OP, Storm-Mathisen J (1987). Anatomical organization of excitatory amino acid receptors and their pathways. TINS.

[b17] Cunha RA, Johansson B, Constantino MD, Sebastifio AM, Fredholm BB (1996). Evidence for high-affinity binding sites for the adenosine A2A receptor agonist [3H]CGS 21680 in the rat hippocampus and cerebral cortex that are different from striatal A2A receptors. Naunyn-Schmiedeberg's ArchPharmacol.

[b18] Dixon CJ, Hall JF, Webb TE, Boarder MR (2004). Regulation of rat hepatocyte function by P2Y receptors: focus on control of glycogen phosphorylase and cyclic AMP by 2-methylthioadenosine 5′-diphosphate. J.Pharmacol. Exp. Ther.

[b19] Duarte JMN, Oliveira CR, Ambrosio AF, Cunha RA (2006). Modification of adenosine A1 and A2A receptor density in the hippocampus of streptozotocin-induced diabetic rats. Neurochem. Int.

[b20] Dunwiddie TV, Masino SA (2001). The role and the regulation of adenosine in the central nervous system. Annu. Rev. Neurosci.

[b21] Franke H, Grummich B, Hartig W, Grosche J, Regenthal R, Edwards RH, Illes P, Krugel U (2006a). Changes in purinergic signal after injury- involvement of glutamatergic mechanisms?. Int. J. Dev. Neurosci.

[b22] Franke H, Illes P (2006). Involvement of P2 receptors in the growth and survival of neurons in the CNS. Pharmacol. Ther.

[b23] Franke H, Krugel U, Grosche J, Heine C, Hartig W, Allgaier C, Illes P (2004). P2Y receptor expression onastrocytes in the nucleus accubens of rats. Neuroscience.

[b24] Franke H, Krügel U, Illes P (2006b). P2 receptors and neuronal injury. Pflugers Arch.

[b25] Franke H, Krugel U, Schmidt R, Grosche J, Reichenbach A, Illes P (2001). P2 receptor-types involved in astrogliosis *in vivo*. Br. J. Pharmacol.

[b26] Fredholm BB (1997). Adenosine and neuroprotection. Int. Rev. Neurobiol.

[b27] Fredholm BB, Abbracchio MP, Burnstock G, Daily JW, Harden TK, Jacobson KA, Leff P, Williams M (1994). Nomenclature and classification of purinoreceptors. Pharmacol. Rev.

[b28] Fredholm BB, Assender JW, Irenius E, Kodama N, Saito N (2003). Synergistic effects of adenosine A_1_ and P2Y receptor stimulation on calcium mobilization and PKC translocation in DDT1 MF-2 cells. Cell. Mol. Neurobiol.

[b29] Fumagalli M, Trincavelli L, Lecca D, Martini C, Ciana P, Abbracchio MP (2004). Cloning, pharmacological characterisation and distribution of the rat G protein-coupled P2Y13 receptor. Biochem. Pharmacol.

[b30] Gao ZG, Blaustein JB, Gross AS, Melman N, Jacobson KA (2003). N6-Substituted adenosine derivatives: selectivity, efficacy, and species differences at A3 adenosine receptors. Biochem. Pharmacol.

[b31] Giuntini J, Giusti L, Lucacchini A, Mazzoni MR (2004). Modulation of A1 adenosine receptor signaling by peroxynitrite. Biochem. Pharmacol.

[b32] Gottlieb M, Matute C (1997). Expression of ionotropic receptor subunits in glial cells of the hippocampal CA1 area following transient forebrain ischemia. J. Cereb. Blood Flow Metab.

[b33] Gundersen HJ, Bendtsen TF, Korbo L, Marcussen N, Moller A, Nielsen K, Nyengaard JR, Pakkenberg B, Sorensen FB, Vesterby A (1988). Some new, simple and efficient stereological methods and their use in pathological research and diagnosis. APMIS.

[b34] Gundersen V, Chaudhry FA, Bjaalie JG, Fonnum F, Ottersen OP, Storm-Mathisen J (1998). Synaptic vesicular localization and exocytosis of 1-aspartate in excitatory nerve terminals: a quantitative immunogold analysis in rat hippocampus. J. Neurosci.

[b35] Gylterud Owe S, Bogen IL, Walaas SI, Storm-Mathisen J, Bergersen LH (2005). Ultrastructural quantification of glutamate receptors at excitatory synapses in hippocampus of synapsin I+II double knock-out mice. Neuroscience.

[b36] Hagberg H, Lehmann A, Sandberg M (1985). Ischemia-induced shift of inhibitory and excitatory amino acids from intra- to extracellular compartments. J. Cereb. Blood Flow Metab.

[b37] Harry GJ, Lefebvre d'Hellencourt C (2003). Dentate gyrus: alterations that occur with hippocampal injury. Neurotoxicology.

[b38] Hoffmann C, Moro S, Nicholas RA, Harden TK, Jacobson KA (1999). The role of amino acids in extracellular loops of the human P2Y1 receptor in surface expression and activation processes. J. Biol. Chem.

[b39] Hollopeter G, Jantzen HM, Vincent D, Li G, England L, Ramakrishnan V, Yang RB, Nurden P, Nurden A, Julius D, Conley PB (2001). Identification of the platelet ADP receptor targeted by antithrombotic drugs. Nature.

[b40] Hourani SMO, Bailey SJ, Nicholls J, Kitchen I (1991). Direct effect of adenylyl 5′-(β-γ-methylene) diphosphonate, a stable ATP analogue, on relaxant P_1_-purinoceptors in smooth muscle. Br. J. Pharmacol.

[b41] Jimenez AI, Castro E, Communi D, Boeynaems JM, Delicato EG, Miras-Portugal MT (2000). Coexpression of several types of metabotropic nucleotide receptors in single cerebellar astrocytes. J. Neurochem.

[b42] Jourdain P, Bergersen LH, Bhaukaurally K, Bezzi P, Santello M, Domercq M, Matute C, Tonello F, Gundersen V, Volterra V (2007). Glutamate exocytosis from astrocytes controls synaptic strength. Nature Neurosci.

[b43] Kawamura M, Gachet C, Inoue K, Kato F (2004). Direct excitation of inhibitory interneurons by extracellular ATP mediated by P2Y1 receptors in the hippocampal slice. J. Neurosci.

[b44] Koizumi S, Fujishita K, Tsuda M, Shigemoto-Mogami Y, Inoue K (2003). Dynamic inhibition of excitatory synaptic transmission by astrocyte derived ATP in hippocampal cultures. Proc. Natl Acad. Sci. USA.

[b45] Lasley RD, Narayan P, Uittenbogaard A, Smart EJ (2000). Activated cardiac adenosine A1 receptors translocate out of caveolae. J. Biol. Chem.

[b46] Lopes LV, Rebola N, Pinheiro PC, Richardson PJ, Oliveira CR, Cunha RA (2003). Adenosine A3 receptors are located in neurons of the rat hippocampus. Neuroreport.

[b47] Lorenzen A, Fuss M, Vogt H, Schwabe U (1993). Measurement of guanine nucleotide-binding protein activation by A1 adenosine receptor agonists in bovine brain membranes: stimulation of guanosine-5′-*O*-(3-[^35^S-thio) -triphosphate binding. Mol. Pharmacol.

[b48] Lorenzen A, Guerra L, Vogt H, Schwabe U (1996). Interaction of full and partial agonists of the A1 adenosine receptor with receptor/G protein complexes in rat brain membranes. Mol. Pharmacol.

[b49] Maemoto T, Finlayson K, Olverman HJ, Akahane A, Horton RW, Butcher SP (1997). Species differences in brain adenosine A1 receptor pharmacology revealed by use of xanthine and pyrazolopyridine based antagonists. Br. J. Pharmacol.

[b50] Masino SA, Diao L, Illes P, Zahniser NR, Larson GA, Johansson B, Fredholm BB, Dunwiddie TV (2002). Modulation of hippocampal glutamatergic transmission by ATP is dependent on adenosine A_1_ receptors. J. Pharmacol. Exp. Ther.

[b51] Mendoza-Fernandez V, Andrew RD, Barajas-Lopez C (2000). ATP inhibits glutamate synaptic release by acting at P2Y receptors in pyramidal neurons of hippocampal slices. J. Pharmacol Exp. Ther.

[b52] Moore DJ, Chambers JK, Murdock PR, Emson PC (2002). Human Ntera-2/D1 neuronal progenitor cells endogenously express a functional P2Y1 receptor. Neuropharmacol.

[b53] Moore D, Chambers J, Waldvogel H, Faull R, Emson P (2000). Regional and cellular distribution of the P2Y1 purinergic receptor in the human brain: striking neuronal localization. J. Comp. Neurol.

[b54] Moran-Jimenez MJ, Matute C (2000). Immunohistochemical localization of the P2Y1 purinergic receptor in neurons and glial cells of the central nervous system. Mol. Brain Res.

[b55] Moro S, Guo D, Camaioni E, Boyer JL, Harden TK, Jacobson KA (1998). Human P2Y1 receptor: molecular modeling and site-directed mutagenesis as tools to identify agonist and antagonist recognition sites. J. Med. Chem.

[b56] Munshi R, Pang IH, Sternweis PC, Linden J (1991). A1 adenosine receptors of bovine brain couple to guanine nucleotide-binding proteins G1, G2, and G0. J. Biol. Chem.

[b57] Murphy KMM, Snyder SH (1982). Heterogeity of adenosine A1 receptor binding in brain tissue. Mol. Pharmacol.

[b58] Nagelhus EA, Veruki ML, Torp R, Haug FM, Laake JH, Nielsen S, Agre P, Ottersen OP (1998). Aquaporin-4 water channel protein in the rat retina and optic nerve: polarized expression in Muller cells and fibrous astrocytes. J. Neurosci.

[b59] Nakata H, Yoshioka K, Kamiya T (2004). Purinergic-receptor oligomerization: implications for neural functions in the central nervous system. Neurotox. Res.

[b60] Neary JT, Kang Y, Willoughby KA, Ellis EF (2003). Activation of extracellular signal-regulated kinase by stretch-induced injury in astrocytes involves extracellular ATP and P2 purinergic receptors. J. Neurosci.

[b61] Newman EA (2003). New roles for astrocytes: regulation of synaptic transmission. TINS.

[b62] Niebauer RT, Gao ZG, Li B, Wess J, Jacobson KA (2005). Signaling of the human P2Y (1) receptor measured by a yeast growth assay with comparisons to assays of phospholipase C and calcium mobilization in 1321N1 human astrocytoma cells. Purinergic Signal.

[b63] Ochiishi T, Chen L, Yukawa A, Saitoh Y, Sekino Y, Arai T, Nakata H, Miyamoto H (1999). Cellular localization of adenosine A1 receptors in rat forebrain: immunohistochemical analysis using adenosine A1 receptor-specific monoclonal antibody. J. Comp. Neurol.

[b64] Ottersen OP, Zhang N, Walberg F (1992). Metabolic compartmentation of glutamate and glutamine: morphological evidence obtained by quantitative immunocytochemistry in rat cerebellum. Neuroscience.

[b65] Palmer RK, Boyer JL, Schachter JB, Nicholas RA, Harden TK (1998). Agonist action of adenosine triphosphates at the human P2Y1 receptor. Mol. Pharmacol.

[b66] Piper AS, Hollingsworth M (1996). ATP and β-γ-methylene ATP produce relaxation of guinea-pig isolated trachealis muscle via actions at P1 purinoceptors. Eur. J. Pharmacol.

[b67] Ponzio TA, Hatton GI (2005). Adenosine postsynaptically modulates supraoptic neuronal excitability. J. Neurophysiol.

[b68] Rathbone MP, Middlemiss PJ, Gysbers JW, Andrew C, Herman MA, Reed JK, Ciccarelli R, Di Iorio P, Caciagli F (1999). Trophic effects of purines in neurons and glial cells. Prog. Neurobiol.

[b69] Rebola N, Pinheiro PC, Oliveira CR, Malva JO, Cunha RA (2003). Subcellular localization of adenosine A_1_ receptors in nerve terminals and synapses of the rat hippocampus. Brain Res.

[b70] Rebola N, Rodrigues RJ, Lopes LV, Richardson PJ, Oliveira CR, Cunha RA (2005). Adenosine A1 and A2A receptors are co-expressed in pyramidal neurons and co-localized in glutamatergic nerve terminals of the rat hippocampus. Neuroscience.

[b71] Rodrigues RJ, Almeida T, Richardson PJ, Oliviera CR, Cunha RA (2005). Dual presynaptic control by ATP of glutamate release via facilitatory P2X1, P2X2/3, and P2X3 and inhibitory P2Y1, P2Y2, and/or P2Y4 receptors in the rat hippocampus. J. Neurosci.

[b72] Rudolphi KA, Schubert P, Parkinson FE, Fredholm BB (1992). Adenosine and brain ischemia. Cerebrovasc. Brain Metab. Rev.

[b73] Sasaki Y, Hoshi M, Akazawa C, Nakamura Y, Tsuzuki H, Inoue K, Kohsaka S (2003). Selective expression of Gi/o-coupled ATP receptor P2Y12 in microglia in rat brain. Glia.

[b74] Swanson TH, Drazba JA, Rivkees SA (1995). Adenosine A1 receptors are located predominantly on axons in the rat hippocampal formation. J. Comp. Neurol.

[b75] Trincavelli ML, Marroni M, Tuscano D, Ceruti S, Mazzola M, Mitro N, Abbracchio MP, Martini C (2004). Regulation of A2B adenosine receptor functioning by tumor necrosis factor a in human astroglial cells. J. Neurochem.

[b76] Vohringer C, Schafer R, Reiser G (2000). A chimeric rat brain P2Y1 receptor tagged with green-fluorescent protein: high-affinity ligand recognition of adenosine diphosphates and triphosphates and selectivity identical to that of the wild-type receptor. Biochem. Pharmacol.

[b77] Volonté C, Amadio S, D'Ambrosi N, Colpi M, Burnstock G (2006). P2 receptor web: Complexity and fine-tuning. Pharmacol. Ther.

[b78] Von Kugelgen I (2006). Pharmacological profiles of cloned mammalian P2Y-receptor subtypes. Pharmacol. Ther.

[b79] Waldo GL, Harden TK (2004). Agonist binding and Gq-stimulating activities of the purified human P2Y1 receptor. Mol. Pharmacol.

[b80] Wang L, Karlsson L, Moses S, Hultgardh-Nilsson A, Andersson M, Borna C, Gudbjartsson T, Jern S, Erlinge D (2002). P2 receptor expression profiles in human vascular smooth muscle and endothelial cells. J. Cardiovasc. Pharmacol.

[b81] Wang BL, Larsson LI (1985). Simultaneous demonstration of multiple antigens by indirect immunofluorescence or immunogold staining. Novel light and electron microscopical double and triple staining method employing primary antibodies from the same species. Histochemistry.

[b82] Wardas J (2002). Neuroprotective role of adenosine in the CNS. Pol. J. Pharmacol.

[b83] Yoshioka K, Hosoda R, Kuroda Y, Nakata H (2002). Hetero-oligomerization of adenosine A1 receptors with P2Y1 receptors in rat brains. FEBS Lett.

[b84] Yoshioka K, Nakata H (2004). ATP- and adenosine-mediated signaling in the central nervous system: purinergic receptor complex: generating adenine nucleotide-sensitive adenosine receptors. J. Pharmacol. Sci.

[b85] Yoshioka K, Saitoh O, Nakata H (2001). Heteromeric association creates a P2Y-like adenosine receptor. Proc. Natl Acad. Sci. USA.

[b86] Zhang FL, Luo L, Gustafson E, Palmer K, Qiao X, Fan X, Yang S, Laz TM, Bayne M, Monsma F (2002). P2Y_13_: identification and characterization of a novel Galphai-coupled ADP receptor from human and mouse. J. Pharmacol. Exp. Ther.

[b87] Zhu Y, Kimelberg HK (2001). Developmental expression of metabotropic P2Y1 and P2Y2 receptors in freshly isolated astrocytes from rat hippocampus. J. Neurochem.

[b88] Zimmermann H (2000). Extracellular metabolism of ATP and other nucleotides. Naunyn Schmiedebergs ArchPharmacol.

